# Heparanase 2 Modulation Inhibits HSV-2 Replication by Regulating Heparan Sulfate

**DOI:** 10.3390/v16121832

**Published:** 2024-11-26

**Authors:** James Hopkins, Ipsita Volety, Farreh Qatanani, Deepak Shukla

**Affiliations:** 1 Department of Ophthalmology and Visual Sciences, College of Medicine, University of Illinois Chicago, Chicago, IL 60612, USA; jjhopkin7@gmail.com (J.H.); ivolet2@uic.edu (I.V.); farreh_qatanani@rush.edu (F.Q.); 2 Department of Microbiology and Immunology, College of Medicine, University of Illinois Chicago, Chicago, IL 60612, USA; 3 Department of Pathology, College of Medicine, University of Illinois Chicago, Chicago, IL 60612, USA

**Keywords:** heparanase 2, herpes simplex virus type 2, heparan sulfate

## Abstract

The host enzyme heparanase (HPSE) facilitates the release of herpes simplex virus type 2 (HSV-2) from target cells by cleaving the viral attachment receptor heparan sulfate (HS) from infected cell surfaces. HPSE 2, an isoform of HPSE, binds to but does not possess the enzymatic activity needed to cleave cell surface HS. Our study demonstrates that HSV-2 infection significantly elevates HPSE 2 protein levels, impacting two distinct stages of viral replication. We show that higher HPSE 2 negatively affects HSV-2 replication which may be through the regulation of cell surface HS. By acting as a competitive inhibitor of HPSE, HPSE 2 may be interfering with HPSE’s interactions with HS. We demonstrate that the enhanced expression of HPSE 2, either via viral infection or plasmid transfection, reduces HPSE’s ability to cleave HS, thereby hindering viral egress. Conversely, low HPSE 2 levels achieved through siRNA transfection allow HPSE to cleave more HS, reducing viral entry. Altogether, we propose a hypothetical model in which the modulation of HPSE 2 impedes HSV-2 replication by regulating HS availability on the cell surface. This dual role of HPSE 2 in viral replication and potential tumor suppression underscores its significance in cellular processes and viral pathogenesis.

## 1. Introduction

Herpes simplex virus type 2 (HSV-2) is a nuclear replicating double-stranded DNA virus, which enters cells via the binding of its envelope glycoproteins, gB and gC, to cell surface heparan sulfate (HS) [1]. HSV-2 is the primary etiological agent of genital herpes, a condition characterized by painful lesions and inflammation in the genital and anal regions [2]. In males, infection typically manifests on the penile shaft or anal area, whereas in females, it predominantly affects the vaginal region, cervix, and perianal area. Moreover, HSV-2 genital infection is associated with an increased risk of both HIV transmission and acquisition [3]. Although HSV-2 can cause ocular and labial infections, these occurrences are less common compared to HSV-1. Currently, there is neither a vaccine nor a cure for HSV-2, and resistance to existing antiviral therapies, such as acyclovir, has been documented [4,5,6]. These antiviral agents, developed over decades ago, primarily target viral DNA replication [7]. Hence, there is a critical need for delineating the mechanisms that drive HSV-2 pathogenesis and identifying new targets for improved interventions.

To understand the host factors that facilitate the viral lifecycle, our study focuses on an HS binding host protein, heparanase 2 (HPSE 2) [8]. HPSE 2 is the only known cell protein that sequesters HS, a ubiquitous cell surface polysaccharide and HSV-2 attachment receptor, by inhibiting heparanase (HPSE) [9]. Unlike HPSE, which cleaves HS, HPSE 2 competitively inhibits HPSE’s HS cleaving activity [10] by targeting the same epitopes with a higher affinity but without any enzymatic activity of its own [11]. HPSE 2 has been recognized as a tumor suppressor in various studies, regulating genes involved in tumor vascularity, fibrosis, cell differentiation, endoplasmic reticulum stress, and apoptosis [12,13,14,15,16,17,18]. Three splice variants of HPSE 2 exist and they contribute to overlapping functions in health and disease.

In recent studies by our lab, we have investigated the pro-viral functions of HPSE in vitro and in vivo models in HSV-1 infections and in vitro models regarding HSV-2 infection [19,20]. In our studies, we found that HS is needed for viral entry but its removal after viral replication by HPSE is required for the optimal release of newly generated virion particles. We also showed that higher levels of HPSE during infection contribute to viral pathogenesis and the induction of a proinflammatory environment. While several past studies have focused on HPSE in viral infections, very little knowledge exists on the role of its competitive inhibitor, HPSE-2. In this study, we show that the overexpression and knockdown of HPSE 2 negatively affects HSV-2 replication, shedding light on the molecular interactions that regulate viral entry and egress from host cells.

## 2. Materials and Methods

### 2.1. Cells and Viruses

Human vaginal epithelial (VK2/E6E7) cells were obtained from ATCC. VK2/E6E7 cells were passaged in Keratinocyte serum-free medium (KSFM) (Gibco/BRL, Carlsbad, CA, USA) supplemented with epidermal growth factor (EGF), bovine pituitary extract (BPE), and 1% penicillin/streptomycin. For convenience, cells in this cell line are referred to as VK2 cells throughout. All infections were inoculated with HSV-2 333 at an MOI of 0.1 or 1 on VK2 cells unless otherwise mentioned. The Vero cell line (African green monkey kidney) was generously given by Dr. Patricia G. Spear (Northwestern University, Chicago, IL, USA) and cultured in DMEM (Gibco, Waltham, MA, USA) with 10% FBS (Sigma, St. Louis, MO, USA) and 1% penicillin/streptomycin (Gibco). OGT 2115 (Tocris Biosciences, Bristol, United Kingdom) was used for heparanase activity inhibition and has been previously described as an HPSE-1 inhibitor [21]. OGT 2115 was used at 10 μM unless otherwise specified. Viruses used were wild-type HSV-2 (333) and HSV-2 (333) GFP [22] and were a kind gift from Dr. Patricia G. Spear (Northwestern University, Chicago, IL, USA). Virus stocks were grown and titered on Vero cells and stored at −80 °C.

### 2.2. Antibodies, Plasmids, and siRNA

HPSE 2 polyclonal antibody (Proteintech, Chicago, IL, USA) was used for Western blot (1:1000), imaging (1:100), and flow cytometry studies (1:100). Anti-human HS monoclonal antibody 10E4 (US Biological, Salem, MA, USA) was used for flow cytometry (1:100) and cell imaging (1:100). GAPDH (Santa Cruz Biotechnology, Dallas, TX, USA) was used for Western blot analysis at dilution of 1:2000. HSV-2 gD [2C10] antibody (abcam, Cambridge, MA, USA) was used for Western blot analysis at a dilution of 1:2000. The HPSE-2a, 2b, and 2c plasmids were provided by Dr. Edward A McKenzie (Manchester Institute of Biotechnology, Manchester, United Kingdom). GS3-HPSE-1 plasmid was a gift from Dr. Israel Vlodavsky (Rappaport Institute, Haifa, Israel). All transfections were performed using Lipofectamine-2000 (Life Technologies, Carlsbad, CA, USA).

### 2.3. Western Blot Analysis

Proteins from samples in this study were collected using radio immunoprecipitation assay (RIPA) buffer (Sigma-Aldrich, St. Louis, MO, USA) according to the manufacturer’s protocol. After gel electrophoresis, membranes were blocked in 5% BSA for 1 h followed by incubation with primary antibody for 1 h, then incubation with respective secondary antibodies (anti-mouse 1:10,000 and anti-rabbit 1:10,000) for 1 h. Protein bands were visualized using the SuperSignal West Femto maximum sensitivity substrate (Thermo Scientific, Waltham, MA, USA) with ImageQuant LAS 4000 biomolecular imager (GE Healthcare Life Sciences, Pittsburgh, PA, USA). The densities of the bands were quantified using ImageJ 1.52a image analysis software (NIH, Bethesda, MD, USA).

### 2.4. PCR and RNA Interference (RNAi)

Trizol (Life Technologies) was used to obtain RNA following the protocol from the manufacturer, then a cDNA Reverse Transcription Kit from Applied Biosystems was used to transcribe to DNA following the protocol of the manufacturer. Fast SYBR Green Master Mix (Applied Biosystems) was used to perform real-time quantitative PCR, using QuantStudio 7 Flex (Applied Biosystems).

The primers used in this study are as follows: HPSE-2 forward primer 5′-CGCCTGTTAGACACACTCTCTGA-3′ and reverse primer 5′-GTCACCACACCTTCAAGCCAA-3′; and GAPDH forward primer 5′-TCCACTGGCGTCTTCACC-3′ and reverse primer 5′-GGCAGAGATGATGACCCTTTT-3′. For small interfering RNA (siRNA) experiments, VK2 E6E7 cells were transfected with human HPSE 2 and diluted in Opti-MEM (Gibco) using Lipofectamine RNAiMAX according to the manufacturer’s instructions. The siRNA sequences si1, si2, si3, and si4 were incubated in cells for 48 h and then infected with HSV-2. The siRNA sequences for human HPSE 2 are as follows.
**siHPSE2 (si1)** –**Forward**: 5′-GAGCACCAAGAACCCAGTCA-3′ **Reverse**: 5′-AAGCGCTTGGAGCTTAGGAA-3′**siHPSE2 (si2)** –**Forward**: 5′-GAGCACCAAGAACCCAGTCA-3′ **Reverse**: 5′-AAGCGCTTGGAGCTTAGGAA-3′**siHPSE2 (si3)** –**Forward**: 5′-GAGCACCAAGAACCCAGTCA -3′ **Reverse**: 5′-AAGCGCTTGGAGCTTAGGAA-3′**siHPSE2 (si4)** –**Forward**: 5′- GAGCACCAAGAACCCAGTCA-3′ **Reverse**: 5′- AAGCGCTTGGAGCTTAGGAA-3′

### 2.5. Flow Cytometry

Measurement of HS and HPSE-1 cell surface expression was performed after HSV-2 333-WT infection. Monolayers of VK2 cells were infected at an MOI of 0.1 or 1 then harvested at different times post-infection (12 hpi, 24 hpi, 36 hpi, and 48 hpi). Cells were harvested and fixed with 4% PFA for 10 min, then incubated with 5% BSA for 1 h followed by incubation with FITC-conjugated anti-HS with a 1:100 dilution in PBS with 1% BSA for 1 h. Cells were then suspended in FACS buffer (PBS, 5% FBS, and 0.1% sodium azide). For detection of HPSE-1 on the cell surface, cells were harvested then fixed with 4% PFA for 4 min, then incubated with 5% BSA for 1 h, and then incubated with primary antibody diluted in PBS with 5% BSA for 1 h, followed by incubation with FITC-conjugated secondary antibody. Cells were then resuspended in FACS buffer. Cells stained with respective FITC-conjugated secondaries only were used as background controls. Entire cell populations were used for the mean fluorescence intensity calculations. At the termination of cellular incubations, cells were collected on ice, washed twice with FACS buffer, and analyzed with a BD Accuri C6 Plus flow cytometer. BD Accuri C6 Plus software and Treestar FlowJo v10.0.7 were used for all flow cytometry data analyses. Briefly, the gating strategy is as follows: (1) live cells were gated using forward scattering (FSC-A) and side scattering (SSC-A); (2) single cells from the live gate were gated using FSC-A and FSC-H gates; and (3) single cells were then plotted on a histogram to obtain counts of FITC-positive populations post, and FlowJo v10.0.7 software was used to obtain MFI counts.

### 2.6. Immunofluorescence Microscopy

VK2 cells were cultured in glass bottom dishes (MatTek Corporation, Ashland, MA, USA). Cells were fixed in 4% PFA for 10 min and permeabilized with 0.1% Triton-X for intracellular labelling, then incubated with 5% BSA for 1 h, primary antibody diluted in PBS with 5% BSA for 1 h, and FITC-conjugated secondary. For HPSE-1 surface staining, the protocol was incubation with 5% BSA for 1 h, then primary antibody diluted in PBS with 5% BSA for 1 h, and then FITC-conjugated secondary. Imaging was performed with a Zeiss Confocal 710 scanning microscope, Germany. Pinhole was set to 1 Airy unit. Fluorescence intensity of images was calculated using Zen software version 3.0.

### 2.7. Plaque Assay

Viral egress titers were measured using a plaque assay [23]. Monolayers of VK2 cells were plated in twelve-well plates and infected with HSV-2 333 virus at an MOI of 0.1 or 1. Media were collected at different time points post-infection and titered on Vero cells. Primary incubation of collected media was performed with DPBS+/+ (Life Technologies) with 1% glucose and 1% heat-inactivated serum for 2 h. Vero cells were then incubated with growth media containing 5% methylcellulose for 72 h, fixed with 100% methanol, and stained with crystal violet solution.

### 2.8. Statistics

Error bars of all figures represent standard error of three independent experiments, unless otherwise specified. Asterisks denote a significant difference as determined by Student’s *t*-test (* *p* < 0.05, ** *p* < 0.01, *** *p* < 0.001, **** *p* < 0.0001, ns, not significant).

## 3. Results

### 3.1. HPSE 2 Is Upregulated in Response to HSV-2 Infection

**Figure 1:**
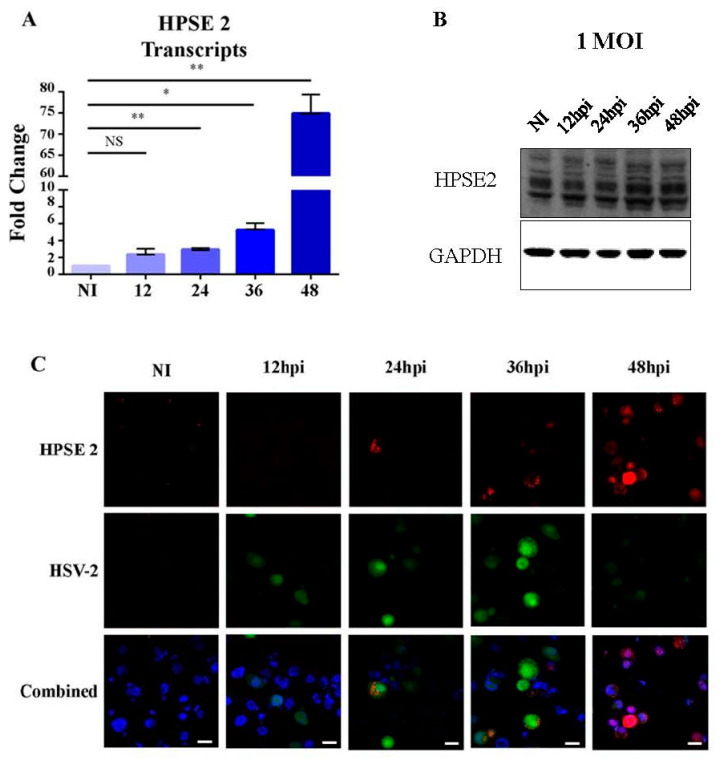
HPSE 2 increases in response to HSV-2 infection. (**A**). Increase in HPSE 2 mRNA levels: vaginal epithelial cells were infected with HSV-2 333, and samples were collected at 0, 12, 24, 36, and 48 h post-infection. The relative fold change over uninfected control is shown. (**B**). Representative Western blot of HPSE 2 expression after HSV-2 333 infection at an MOI of 1 with samples taken at 0, 12, 24, 36, and 48 h post-infection. (**C**). Representative immunofluorescence microscopy images of HPSE 2 stain. HSV-2 333 GFP was used to infect cells at a MOI of 0.1, then images were taken at 0, 12, 24, 36, and 48 h post-infection. The top row is HPSE 2, shown in red; the middle row is HSV-2 GFP; and the last row includes Hoechst, shown in blue, HPSE 2 stain, shown in red, and HSV-2, shown in green. Scale bar, 40 μm. Asterisks on plotted graph denote a significant difference as determined by Student’s *t*-test (* *p* < 0.05, ** *p* < 0.01, NS: not significant).

To understand how HSV-2 infection modulates HPSE 2 levels, we infected an established cell type that is a normal target for the virus, human vaginal epithelial cells (VK2), with HSV-2 at a multiplicity of infection (MOI) of 0.1. We observed a large and significant upregulation of HPSE 2 transcripts starting at 24 hpi through 48 hpi quantified as a 70-fold increase (Figure 1A). The increase in mRNA transcripts corresponded to an increase in HPSE 2 protein levels after infection (with an MOI of 1) from 12 hpi through 48 hpi. Note that three separate bands denoting HPSE 2 splice variants a, b, and c can be distinguished (Figure 1B). Also, by immunofluorescence microscopy after staining for HPSE 2 after infection with HSV-2 333 GFP, which produces GFP on a CMV promoter upon infection, we were able to see an increase in the level of infection that agrees with our Western blot data (Figure 1C).

### 3.2. Overexpression of HPSE 2 Inhibits HSV-2 Replication

**Figure 2:**
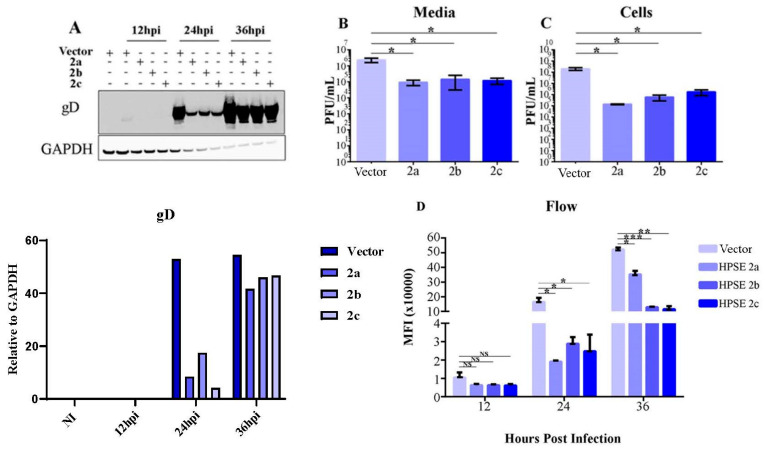
Overexpression of HPSE 2 leads to lower viral replication (**A**). Representative Western blot of expression of viral protein gD after overexpression of HPSE 2 (upper panel). The quantified protein levels of gD are plotted, indicating decreased gD protein levels when isoforms are expressed compared to vector control (lower panel). VK2 cells were infected with HSV-2 333 24 h after transfection with plasmids overexpressing HPSE 2 a, b, and c and a control. Samples were collected at 0 for the control and 12, 24, and 48 h post-infection for all categories. (**B**). Quantification of plaque assay of media collected from cells that were overexpressing HPSE 2 a, b, and c and then were infected with HSV-2 at an MOI of 1. Samples were collected at 24 h post-infection. (**C**). Quantification of plaque assay of cell lysates collected from cells that were overexpressing HPSE 2 a, b, and c and then were infected with HSV-2 at an MOI of 1. Samples were collected at 24 h post-infection. (**D**). Quantification of infection after overexpressing HPSE 2 a, b, and c flow cytometry experiments, with samples collected 12, 24, and 36 h post-infection. All plotted results are presented as mean ± SEM of three independent experiments (n = 3). Asterisks denote a significant difference as determined by Student’s *t*-test; (* *p* < 0.05, ** *p* < 0.01, *** *p* < 0.001, NS: not significant).

In our investigation, we observed that the expression of HPSE 2 varied during HSV-2 infection. To determine whether the further modulation of HPSE 2 could influence viral proliferation, we overexpressed all three splice variants (a, b, and c) of HPSE 2 in VK2 cells, followed by infection with HSV-2 strain 333 at an MOI of 1. Initially, we assessed the expression of a viral late gene product, glycoprotein gD, known for its abundance, using a Western blot analysis. Our results indicated a substantial decrease in gD levels across all time points and in cells overexpressing each splice variant compared to the empty vector (Vector) control (Figure 2A). This finding was unexpected, given that only the HPSE 2c variant is known to localize to the cell surface and interact with HS. Subsequently, we performed a plaque assay on the media from infected cells plated without methylcellulose to allow for the release and spread of extracellular virions. This assay revealed a significant reduction in viral titers in all three-splice variant-overexpressing cells compared to the empty vector control (PcDNA), consistent with the Western blot results (Figure 2B).

We further evaluated the intracellular viral load by conducting a plaque assay on cell lysates from VK2 cells infected with HSV-2 at an MOI of 1. The cells were collected 24 h post-infection and sonicated to release the intracellular virus. The assay showed a marked decrease in viral titers in all three-splice variant-overexpressing cells relative to the control, aligning with the results from the extracellular virus plaque assay and the Western blot (Figure 2C). Lastly, we infected VK2 cells at an MOI of 0.1 with HSV-2 333 GFP following the overexpression of the three HPSE 2 splice variants. At 12, 24, and 36 hpi, we conducted flow cytometry to measure fluorescence in infected cells. The data indicated reduced fluorescence in HPSE 2-overexpressing cells compared to the PcDNA control (Figure 2D). Collectively, these results suggest that the overexpression of HPSE 2 negatively impacts HSV-2 replication, corroborating our findings from the Western blot, plaque assay, and flow cytometry analyses.

### 3.3. Silencing HPSE 2 Curbs HSV-2 Infection

**Figure 3:**
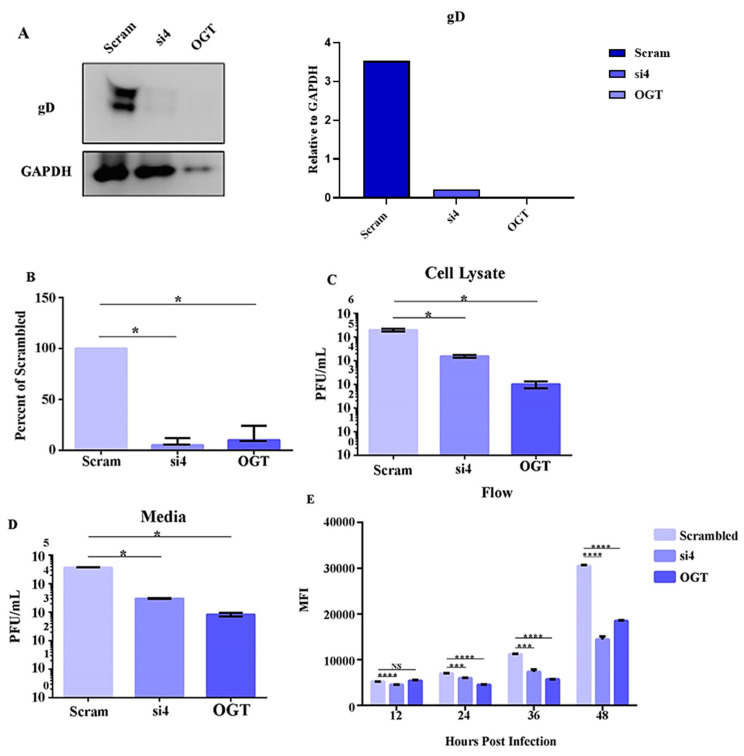
Knockdown of HPSE leads to lowerHSV-2 replication (**A**). Representative Western blot of expression of viral protein gD after knockdown of HPSE 2 and treatment with OGT 2115 after infection with HSV-2 333. The samples were collected 24 h post-infection (left panel). The quantified protein levels of gD are plotted, indicating decreased gD protein levels when isoforms are expressed compared to vector control (right panel). (**B**). Quantification of Western blot of expression of viral protein gD after knockdown of HPSE 2. (**C**). Quantification of a plaque assay of cell lysate collected from HPSE 2 knockdown cells or cells treated with OGT 2115, which then were infected with HSV-2 at an MOI of 1. Samples were collected 24 h post-infection. (**D**). Quantification of a plaque assay of media collected from HPSE 2 knockdown cells or cells treated with OGT 2115, which then were infected with HSV-2 at an MOI of 1. Samples were collected 24 h post-infection. (**E**). Quantification of infection after treatment with OGT 2115 or HPSE 2 knockdown in flow cytometry experiments. All plotted results are presented as mean ± SEM of three independent experiments (n = 3). Asterisks denote a significant difference as determined by Student’s *t*-test; (* *p* < 0.05, *** *p* < 0.001, **** *p* < 0.0001, NS: not significant).

Our investigation so far has demonstrated that HSV-2 infection leads to an increase in HPSE 2 levels, and that the overexpression of HPSE 2 has a detrimental effect on HSV-2 replication. To further explore the influence of reduced HPSE 2 levels on viral proliferation, we designed a small interfering RNA (siRNA)-mediated knockdown of HPSE 2. We synthesized four siRNAs targeting HPSE 2, named si1, si2, si3, and si4, along with a scrambled siRNA as a control. A Western blot analysis identified si4 as the most effective in reducing HPSE 2 protein levels (data not included). Following HPSE 2 knockdown with si4, VK2 cells were infected with HSV-2 strain 333 at an MOI of 0.1. Additionally, control cells treated with either scrambled siRNA (for 12 h as per the manufacturer’s protocol) or cells exposed to the HPSE inhibitor OGT 2115 (at two hpi) for a total of 12 h were used. We first assessed the levels of the abundant viral protein glycoprotein D (gD) using a Western blot analysis. To our surprise, the results showed a significant decrease in gD production in both HPSE 2 knockdown and OGT 2115-treated cells compared to cells treated with scrambled siRNA (Figure 3A). The quantification of the Western blot data confirmed a significant reduction in gD expression in HPSE 2 knockdown and OGT 2115-treated cells (Figure 3B), which was unexpected given that the overexpression of HPSE 2 previously had inhibited HSV-2 infection.

Next, we measured the amount of infectious virus produced, both intracellularly and extracellularly, using plaque assays. The results revealed significantly lower viral titers in both HPSE 2 knockdown and OGT 2115-treated cells, consistent with the Western blot findings (Figure 3C,D). Finally, we infected VK2 cells with HSV-2 333 GFP and measured the mean fluorescence intensity at 12, 24, 36, and 48 hpi as an indicator of infection levels. The results showed a significantly lower level of infection in HPSE 2 knockdown and OGT 2115-treated cells at all time points, corroborating the plaque assay and Western blot data (Figure 3E). These findings indicate that reduced HPSE 2 levels negatively affect HSV-2 replication, highlighting the complex role of HPSE 2 in the viral lifecycle.

### 3.4. Effect of Modulation of HPSE 2 on Cell Surface HS 

**Figure 4:**
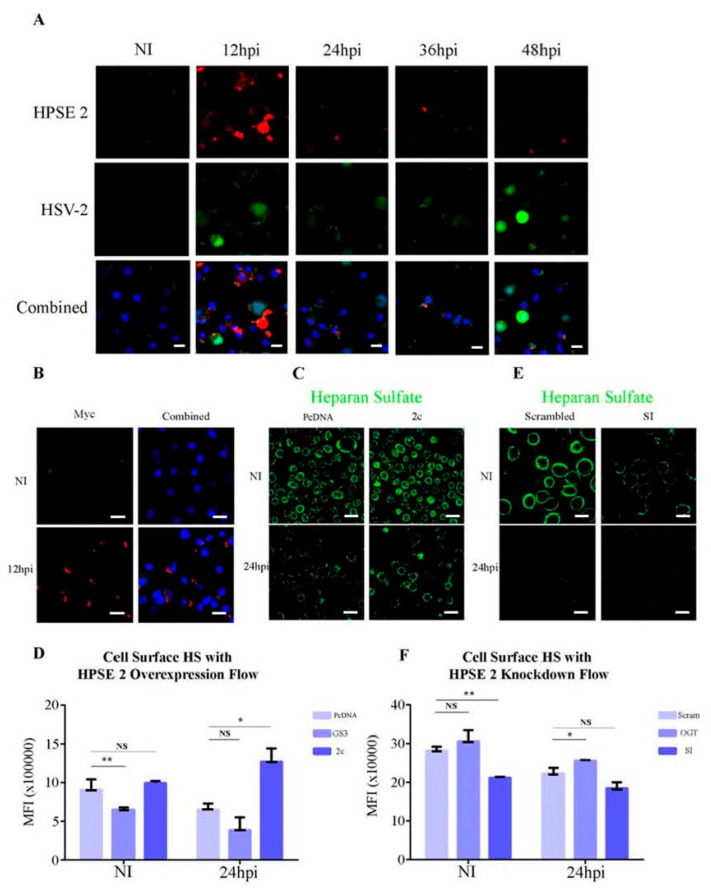
Mechanism of HPSE 2 inhibition of HSV-2 infection. (**A**). Representative immunofluorescent microscopy images of HPSE 2 surface stain. HSV-2 333 GFP was used to infect cells at an MOI of 0.1, then images were taken 0, 12, 24, 36, and 48 h post-infection. The top row is HPSE 2 in red, the middle row is HSV-2 in GFP, and the last row includes Hoechst in blue, HPSE 2 stain in red, and HSV-2 in green. (**B**). Representative immunofluorescent images of surface HPSE 2c-myc stain. HSV-2 333 was used to infect cells at an MOI of 1 for 12 h. Upper left is myc stain only in uninfected sample, upper right is Hoescht and myc stain merged for uninfected sample, lower left is myc stain only for infected sample 12 h post-infection, and lower right is Hoechst and myc stain merged for infected sample 24 h post-infection. (**C**). Representative immunofluorescent images of surface heparan sulfate stain. HSV-2 333 was used to infect cells at an MOI of 1 for 24 h. Left column shows control cells, with uninfected cells on top and infected cells on the bottom. Right column shows cells overexpressing HPSE 2c, with uninfected cells on top and infected cells on the bottom. (**D**). Quantification of cell surface heparan sulfate flow cytometry experiments by flow with cells overexpressing HPSE 2. (**E**). Representative immunofluorescent images of surface heparan sulfate stain. HSV-2 333 was used to infect cells at an MOI of 1 for 24 h. Left column shows control cells, with uninfected cells on top and infected cells on the bottom. Right column shows cells with HPSE 2c knockdown, with uninfected cells on top and infected cells on the bottom. (**F**). Quantification of cell surface heparan sulfate flow cytometry experiments used cells with HPSE 2 knockdown. Scale bar, 40 μm. All plotted results are presented as mean ± SEM of three independent experiments (n = 3). Asterisks denote a significant difference as determined by Student’s *t*-test; (* *p* < 0.05, ** *p* < 0.01, NS: not significant).

After establishing that both the overexpression and knockdown of HPSE 2 negatively affect HSV-2 replication, we aimed to determine if these effects are mediated through HPSE 2′s regulation of cell surface heparan sulfate (HS). To investigate this, we first examined whether HPSE 2 localization to the cell surface changes during infection. Using confocal microscopy at 63× magnification, we stained for HPSE 2 at the cell surface of VK2 cells infected with HSV-2 at an MOI of 0.1 (Figure 4A). We observed a significant increase in the cell surface localization of HPSE 2 12 h post-infection, which decreased by 24 hpi and remained low to 48 hpi. Next, we focused on HPSE 2c, the only splice variant known to localize to the cell surface. We overexpressed an HPSE 2c-myc construct in VK2 cells, infected them with HSV-2 at an MOI of 1, and conducted cell surface straining for myc to visualize only the cell surface localization of our construct with and without infection. Our results indicated a significant change in the localization of myc and, consequently, HPSE 2c upon infection, confirming that HPSE 2c localizes to the cell surface during infection (Figure 4B).

We then examined whether the overexpression of HPSE 2c can sequester HS at the cell surface during infection. Immunofluorescence microscopy showed that overexpressed HPSE 2c retained HS at the cell surface to 24 hpi (Figure 4C). This finding contrasts with previous publications from our lab, which demonstrated a substantial decrease in HS cell surface localization 24 h post-infection. Lastly, we assessed the ability of HPSE 2 splice variants to sequester HS at the cell surface. We conducted flow cytometry to measure for cell surface HS after overexpressing HPSE2c alongside the positive control GS3-HPSE, which is a constitutively active HPSE1 construct previously shown to cleave cell surface HS [19]. We observed that compared to the PcDNA vector, HPSE2c overexpression showed higher HS cell surface levels post-infection (Figure 4D). When we treated our cells with OGT 2115 to inhibit HPSE1 followed by infection with HSV-2 strain 333 for 24 h, we observed a reduction in HS staining in the non-infected, HPSE2-silenced cells and negligible differences between the PcDNA control and siHPSE2 in the infected conditions (Figure 4F). Finally, silencing HPSE2 decreased the cell surface localization of HS (Figure 4E). These findings suggest that the regulatory effects of HPSE 2 on HSV-2 replication are mediated through its modulation of cell surface HS, with significant changes in HPSE2 localization and HS cell surface localization observed during the infection.

## 4. Discussion

In this study, we have elucidated the novel role of HPSE 2 in the context of HSV-2 infection, demonstrating its critical involvement in regulating viral replication through the modulation of HS dynamics at the cell surface. Contrary to our initial expectations based on previous findings with HSV-1 and HSV-2 infections, in which elevated HPSE levels were associated with enhanced viral replication, our current data reveal that HPSE 2 overexpression or knockdown leads to consistent reductions in HSV-2 replication, despite its ability to repress HPSE’s enzymatic activity [11,24].

Our findings suggest that HPSE 2 plays a dual role in HSV-2 pathogenesis. Consistent with previous reports, HPSE 2 first appears to sequester HS at the cell surface by competitively binding to HS epitopes with a higher affinity, thereby limiting the availability of HS for viral binding and entry [25,26,27,28]. Secondly, in the absence of HPSE 2, HPSE activity is increased, resulting in the enhanced cleavage of HS and facilitating viral egress from infected cells. This dual mechanism underscores the intricate balance between HPSE 2-mediated HS sequestration and HPSE-driven HS cleavage in regulating viral replication dynamics.

Furthermore, our proposed model integrates these observations, proposing that optimal HPSE 2 levels are crucial for HSV-2 pathogenesis. Insufficient HPSE 2 prior to viral binding may lead to inadequate HS presentation at the cell surface, impairing viral entry. Conversely, an early high expression of HPSE 2 during infection may shield HS, thereby impeding viral egress by maintaining higher HS levels at the cell surface. Moving forward, elucidating the precise molecular mechanisms through which HPSE 2 regulates HS availability and HPSE activity during HSV-2 infection will be essential. These insights could not only deepen our understanding of viral pathogenesis but also uncover potential therapeutic targets for modulating HS dynamics to mitigate HSV-2 replication and associated clinical manifestations. 

## Data Availability

Further information and requests for resources and reagents should be directed to and will be fulfilled by the lead contact, Deepak Shukla (dshukla@uic.edu).
